# Antibacterial activity and mechanism of Stevia extract against antibiotic-resistant *Escherichia coli* by interfering with the permeability of the cell wall and the membrane

**DOI:** 10.3389/fmicb.2024.1397906

**Published:** 2024-09-18

**Authors:** Xu Chen, Lan-Kun Yi, Yu-Bin Bai, Ming-Ze Cao, Wei-Wei Wang, Zi-Xuan Shang, Jia-Jing Li, Mei-Li Xu, Li-Fei Wu, Zhen Zhu, Ji-Yu Zhang

**Affiliations:** ^1^College of Life Science and Food Engineering, Hebei University of Engineering, Handan, China; ^2^Key Laboratory of New Animal Drug Project of Gansu Province, Key Laboratory of Veterinary Pharmaceutical Development of the Ministry of Agriculture, Lanzhou Institute of Husbandry and Pharmaceutical Sciences of CAAS, Lanzhou, China; ^3^Chenguang Biological Technology Group Co, Ltd., Handan, China

**Keywords:** ICAC, *E. coli*, antibacterial activity and mechanisms, cell wall, membrane

## Abstract

Natural plant-derived compounds with broad-spectrum antimicrobial activity have become an effective strategy against multidrug-resistant bacteria. The present study was designed to compare the antibacterial activity of six chlorogenic acid (CA) isomers extracted from stevia and investigated the underlying antibacterial mechanisms involved. The results indicated that isochlorogenic acid C (ICAC) exhibited the strongest antibacterial activity against the tested bacteria, especially *E. coli*, at a 2 mg/mL minimum inhibitory concentration (MIC) and 8 mg/mL minimum bactericidal concentration (MBC). At the MBC, ICAC inhibited 72.66% of the clinical multidrug-resistant strains. Scanning electron microscopy (SEM) revealed that ICAC induced considerable morphological alterations in *E. coli* ATCC25922 and C4E2. The significant increase in the activity of extracellular alkaline phosphatase (AKP) indicated that ICAC damages the permeability of the bacterial cell wall. Additionally, the intracellular membrane (IM) permeability and the content of lipopolysaccharide (LPS), a main component of the outer membrane (OM), were determined. The significant decrease in LPS content and increased leakage of intracellular proteins and K^+^ from *E. coli* indicated that ICAC could induce the exfoliation of OM and disrupt IM permeability, resulting in the loss of barrier function. The uptake of propidium iodide (PI), a compromised cell membrane nucleic acid stain, and confocal laser scanning microscopy (CLSM) further demonstrated that ICAC disrupted IM integrity. Moreover, the bactericidal effect and damage to bacterial microstructural function occurred in a dose-dependent manner. These data demonstrate that ICAC has excellent antibacterial activity and is a promising approach for overcoming the antibiotic resistance of pathogenic bacteria.

## 1 Introduction

Pathogenic bacteria are a serious threat that has increased morbidity and mortality related to infectious illnesses in animals and humans worldwide (El-Saadony et al., 2022; Li et al., 2022). Antimicrobial therapy is currently the most effective means to control bacterial infection and has played an irreplaceable role in the past hundred years since its discovery (Maillard et al., 2021). Antibiotics are designed to kill bacteria by inhibiting biological processes essential for survival (Liu et al., 2022). However, antibiotics are similar to double-edged swords. While playing a good therapeutic role, they also cause problems that people cannot predict in advance, such as “3Rs” (resistance, residual, resurgence), especially the long-term and massive use of nonmedical antibiotics (Youngquist et al., 2016; Bhardwaj et al., 2022). The widespread use of antibiotics has placed bacterial pathogens under intense pressure to evolve new survival mechanisms, resulting in the natural selecting of drug-resistant bacteria (Liu et al., 2022). Infections due to antibiotic-resistant organisms are increasing in prevalence and represent a major public health threat. Antibiotic overuse is a major driver of this epidemic (Chiotos et al., 2019).

The development of new antimicrobial agents that can even kill more bacteria has long been considered the appropriate response to the growing threat of antimicrobial-resistant infections. However, the time period between the introduction of a new antibiotic and the emergence of resistance among bacterial pathogens is becoming increasingly shorter, which will lead to the “postantibiotic era” (Yu et al., 2020).

Plant-derived antibacterial agents such as alkaloids, flavonoids, polyphenols, fatty acids, terpenes and other compounds have attracted increasing amounts of attention because of their high safety and abundance of resources (Kokoska et al., 2019; Liu et al., 2021). Phenolic compounds produced in most higher plants could be used for antibacterial, antifungal, antiviral, anti-inflammatory and other biological activities and might significantly reduce the danger of serious health disorders (Ulanowska and Olas, 2021; Alavi et al., 2022). Extensive *in vitro* antimicrobial activities and possible modes of action have been reported for a variety of gram-positive and gram-negative bacterial pathogens, that is, *Salmonella enteritidis*, *Listeria monocytogenes*, *Staphylococcus aureus* and *E. coli* (Vazquez-Armenta et al., 2022; Morshdy et al., 2023). Therefore, increasing attention has been given to these compounds in recent years.

CA, a phenolic compound formed by various herbs, including coffee, tea leaves, honeysuckle, and beans, is recognized as the “plant gold” for its antimicrobial activities (Yang et al., 2022). Classical antibiotics target a specific reaction, whereas natural antimicrobial compounds such as plant polyphenols inhibit several different groups of biomolecules in a pathogen (Xiang et al., 2022). Therefore, resistance to such compounds is unlikely to develop, which makes them attractive antibacterial agents. Previous studies have shown that CA can destroy the bacterial cell wall, change membrane permeability, inhibit the activity of efflux pumps, destroy biofilms and other functional mechanisms (Yang et al., 2022; Samreen Qais and Ahmad, 2023; Wang et al., 2023). However, the antibacterial activity and mechanism of its isomers are limited. Therefore, the objective of this study was to investigate the *in vitro* antibacterial activity and potential mechanism of action of CA and its isomers extracted from stevia against *E. coli*.

## 2 Materials and methods

### 2.1 Raw materials and bacterial strains

CA and its isomers were obtained from stevia via initial extraction, extraction and purification. All the extracts were dissolved in dimethyl sulfoxide (DMSO) purchased from the Beijing Solarbio Science & Technology Co. Ltd. (Beijing, China). Other reagents were of analytical or spectral grade. All the antibiotic solutions were sterilized using 0.22 μm filters prior to use (Jiangsu Green Union Science Instrument, Jiangsu, China).

The *E. coli* strain ATCC 25922 used for quality control was obtained from China General Microbiological Culture Collection Center (Beijing, China). The clinical test strains were multidrug resistant (MDR) *E. coli* isolated from chick livers by the Hebei University of Engineering, Handan. Among them, C4E2 is resistant to TGC and carries the tet(X4) gene. All test strains were activated in Luria–Bertani (LB) nutrient agar (Haibo Biological Technology, Qingdao, China) for 24 h in a 37°C constant temperature incubator. Before use, the bacteria were cultured in LB broth (Haibo Biological Technology, Qingdao, China) for 6 h with constant shaking (250 rpm) at 37°C.

### 2.2 Susceptibility testing

The MICs of the extracts were determined using broth microdilution according to the method described by Clinical and Laboratory Standards Institute (CLSI) (Sweeney et al., 2018). Briefly, serial twofold dilutions of the extracts were prepared and added to sterile 96-well plates, and DMSO was used as a control. The different strains were subsequently inoculated at an initial concentration of 10^6^ CFU/mL. Then, the OD600 was determined after 16 h of incubation. The lowest concentration at which the extract inhibited the growth of the strain was designated the MIC.

The MBC was determined by spreading 100 μL of the broth from clear wells onto MHA plates and incubating for 24 h. The lowest concentration of an antimicrobial agent at which all the cells were killed was defined as the MBC. The experiment was performed in triplicate (El Mihyaoui et al., 2023).

### 2.3 Time–kill kinetic assays

Time–kill kinetic assays for six chlorogenic acid isomers were performed following CLSI methodology (Blais et al., 2022) with slight modifications. The bacteria were inoculated in the early log phase and diluted to a final cell density of 10^6^ CFU/mL, after which the growth of the bacteria was investigated for 24 h. In addition, the strains were incubated with 0, 1/4, 1/2, 1 and 2 × MIC ICAC to analyze the effect of the extract concentration on antibacterial activity.

After each sampling, 10-fold serially diluted in MHB was added, and the mixture was applied directly to MHA plates. Colonies were counted after 16 h of incubation. Based on these counts, CFU/mL was calculated and plotted as Log10 CFU/mL vs. time to generate a time–kill curve (Vila-Farres et al., 2023).

### 2.4 Scanning electron microscopy

The changes in the microstructure and morphology of *E. coli* after treatment with ICA were imaged via SEM (Chen et al., 2022). The log-phase *E. coli* suspension was centrifuged, and the precipitated bacteria were suspended in PBS (0.1 M, pH 7.2). Bacteria were then treated with ICA at various concentrations for 1 h. Then, the cells were fixed with 2.5% glutaraldehyde, washed with PBS, dehydrated in a series of ethanol solutions and permeated with white resin. The morphologies of the *E. coli* cells were ultimately observed via SEM (JSM-6701F, Hitachi, Japan).

### 2.5 Cell wall permeability

*Escherichia coli* cells in the logarithmic growth phase were treated with 0, 1/4, 1/2, 1 and 2 × MIC ICA and then cultured for 6 h, centrifuged and the supernatant was taken for AKP detection. Add 30 μL of supernatant, 50 μL of buffer, and 50 μL of matrix to the 96-well plate, and mix thoroughly in a 37°C water bath for 15 min. Finally, add 150 μL of chromogenic reagent, shake gently to mix. The absorbance values were measured at OD520 nm on a full-wavelength microplate reader. The changes in AKP activity were used to evaluate the effects of ICA on the bacterial cell wall (Gu et al., 2023).

### 2.6 OM permeability

The OM permeability of *E. coli* was determined through change in LPS levels. *E. coli* were cultured to the logarithmic growth stage and treated with ICAC according to the instructions for the AKP test. Then, LPS was extracted from the cultures according to previous methods (Su et al., 2019). The content of LPS was determined by an LPS Elisa Kit (Mlbio, Shanghai, China).

### 2.7 IM permeability

#### 2.7.1 Extracellular protein and K^+^ determinations

The IM permeability of *E. coli* was determined by detecting the release of extracellular proteins and K^+^ efflux. To detect the release of extracellular proteins, the cells were treated with 0, 1/4, 1/2, 1 and 2 × MIC ICA. After ICA treatment, the supernatants were collected, and the extracellular protein levels were detected via a protein quantitation kit (Beyotime, China). The amount of extracellular protein in each sample was obtained by using the standard protein (Sun et al., 2020).

K^+^ efflux was determined as previously described (Lou et al., 2011) with minor modifications. Briefly, *E. coli* ATCC25922 and C4E2 cells were cultured to the logarithmic phase, and 0, 1/4, 1/2, 1 and 2 × MIC ICA was subsequently added to each bacterial suspension for 6 h. After filtration through a microfiltration membrane, the supernatant was collected. K^+^ efflux in *E. coli* cells was determined using a biochemical assay reagent (Elabscience, China).

#### 2.7.2 Measurement of IM integrity with an automatic microplate

A membrane damage assay was used to investigate the effect of ICAC on *E. coli* IM integrity. Damage to the IM was measured using an automatic microplate reader (EnSpire, PerkinElmer, United States) as described by Shi et al. (2022) with some modifications. *E. coli* ATCC25922 and C4E2 were cultured to the logarithmic phase, adjusted to 1.5 × 10^8^ CFU/mL, incubated with propidium iodide (PI) staining solution at a final PI concentration of 15 μM, and then incubated at 37°C for 15 min in the dark. After incubation, 50 μL of culture was added to each well on a black polystyrene microtiter plate. The fluorescence of the stained cells was measured at 535 nm wavelength excitation and 620 nm emission every 2 min for 15 min. Then, 1/4, 1/2, 1 or 2 × MIC ICA was rapidly added, and the fluorescence was measured every 2 min for 1 h.

### 2.8 Analysis of IM integrity with CLSM

To verify the damage to the inner cell membrane, we further detected the cells using CLSM (LSM800, Zeiss, Jena, Germany) as described in a previous report with some modifications (Li et al., 2023). After treatment with various concentrations of ICA, the *E. coli* were collected and washed three times with PBS. Afterward, the cells were dyed with 100 μM carboxyfluorescein diacetate (cFDA) and 15 μM PI in the dark at 25°C for 15 min and then washed. The washed samples were dropped onto a glass slide, covered with a coverslip, and subsequently observed via CLSM.

### 2.9 Statistical analysis

Statistical analysis was carried out through one-way analysis of variance using SPSS software with the Pearson correlation coefficient option (version SPSS26.0). *p* < 0.05 was considered to indicate statistical significance. All the experiments were performed at least in triplicate, and the data are presented as the means ± SDs.

## 3 Results

### 3.1 CA isomers in Stevia

A total of 6 CAisomers were found in stevia: 3-caffeoylquinic acid (3-CA), 4-caffeoylquinic acid (4-CA), 5-caffeoylquinic acid (5-CA), 3,5-dicaffeoylquinic acid (ICAA), 3,4-dicaffeoylquinic acid (ICAB) and 4,5-dicaffeoylquinic acid (ICAC). The chemical structure of the materials are presented in Supplementary Figure S1.

### 3.2 Antibacterial activity

The antibacterial potential of the CA isomers was tested against six microorganisms, and the MIC values are shown in Table 1. The results indicated that all six CA isomers had certain antibacterial effects on all of the tested strains but were more effective against gram-negative bacteria than against gram-positive bacteria. A comparison of the sensitivities of the bacterial strains to the extracts indicated that the strongest inhibitory effect was against *E. coli*. Among them, 3-CA and ICAC were found to have the maximum antibacterial activity against *E. coli* ATCC25922 and C4E2, with MICs of 2 mg/mL and 4 mg/mL, respectively.

**Table 1:** MIC values of chlorogenic acid isomers against six microorganisms.

Strains	MIC of six chlorogenic acid isomers (mg/mL)					
	3-CA	4-CA	5-CA	ICAA	ICAB	ICAC
*E. coli* (ATCC25922)	2	4	4	4	4	2
*E. coli* (C4E2)	4	16	8	8	8	4
*S. flexneri* (301)	4	8	8	16	16	4
*S. aureus* (ATCC25933)	8	16	–	32	8	8
*B. subtili* (ATCC6633)	–	–	16	–	16	8
*P. aeruginosa* (ATCC15442)	16	16	16	32	8	4

“–”: MIC > 32 mg/mL.

The results of the MBC and the ratio of the MBC/MIC for CA isomers against *E. coli* ATCC25922 and C4E2 are shown in Table 2. Compared with the other four isomers, 3-CA and ICAC had minimum MBCs against ATCC25922. However, ICAC had stronger bactericidal activity and a lower MBC/MIC ratio against multidrug-resistant C4E2.

**Table 2:** MBC and MBC/MIC of chlorogenic acid isomers against *E. coli* ATCC25922 and C4E2.

Strains	MBC (mg/mL) and MBC/MIC value											
	3-CA		4-CA		5-CA		ICAA		ICAB		ICAC	
	MBC	MBC/MIC	MBC	MBC/MIC	MBC	MBC/MIC	MBC	MBC/MIC	MBC	MBC/MIC	MBC	MBC/MIC
ATCC25922	8	4	32	8	16	4	16	4	16	4	8	4
C4E2	16	4	64	2	32	4	32	4	32	4	16	4

**Figure 1:**
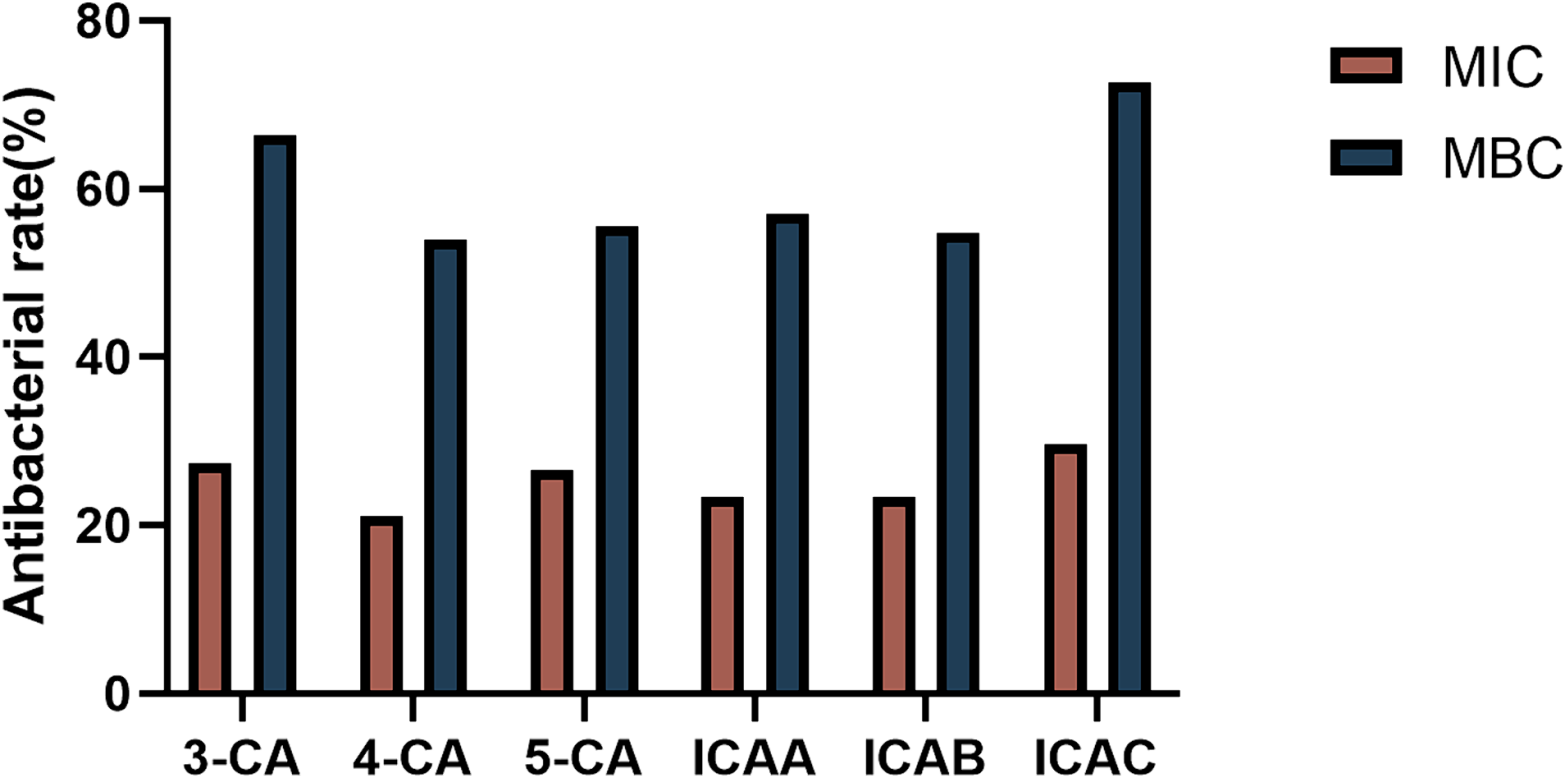
The bacteriostatic ability of CA isomers against 128 multidrug-resistant clinically isolated *E. coli* strains at the MIC and MBC.

Based on the MIC and MBC results, we further investigated the antibacterial activities of different CA isomers against 128 MDR clinically isolated *E. coli* strains. The antibacterial activity at the MBC was obviously greater than that at the MIC (Figure 1). ICAC had the highest bacteriostatic ability and inhibited 72.66% of the test strain at 8 mg/mL ICAC. Hence, the ICAC was selected for further investigation.

### 3.3 Bacterial time–kill kinetic assay

**Figure 2:**
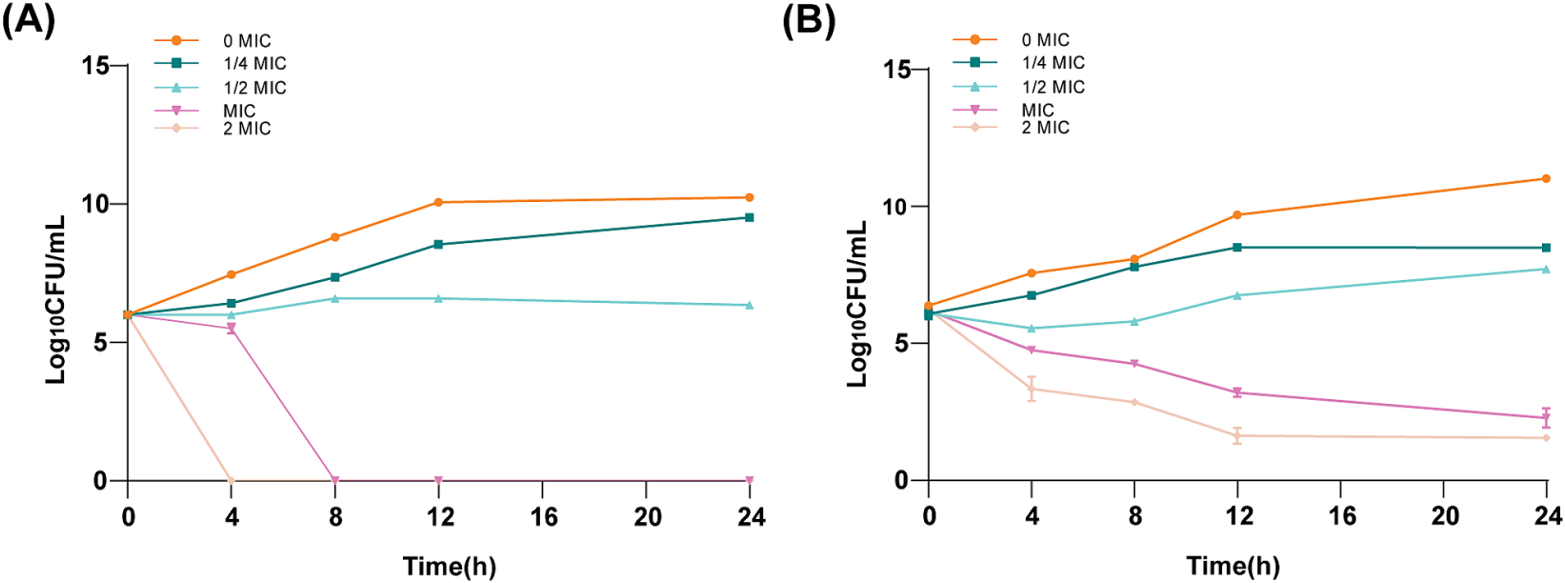
Time–kill kinetic assay of ICAC at a concentration of 0–2× the MIC against ATCC25922 **(A)** and C4E2 **(B)**. The MIC for ATCC25922 was 2 mg/mL, and the MIC for *C4E2* was 4 mg/mL. The results from all the experiments are presented as the means ± SDs of three replicates.

Time–kill kinetic investigations demonstrated a sustained bactericidal effect of ICAC against ATCC 25922 (Figure 2A) and C4E2 (Figure 2B). A low concentration of ICAC (1/4 and 1/2 MIC) partially inhibited the growth of *E. coli*. In addition, the cells exposed to a low concentration of ICAC had recovered and were comparable to the control cultures not exposed to drugs after 24 h of treatment. Furthermore, the inhibitory effect on *E. coli* increased with increasing ICAC concentration. The bactericidal endpoints for ATCC 25922 at 2× and 1× MIC were achieved at the 4 and 8 h time points, respectively. At those concentrations, the growth of C4E2 was also significantly inhibited, and no regrowth was observed. Therefore, the bactericidal activity of ICAC was concentration dependent and time-dependent for both strains.

### 3.4 Cell morphology analysis

**Figure 3:**
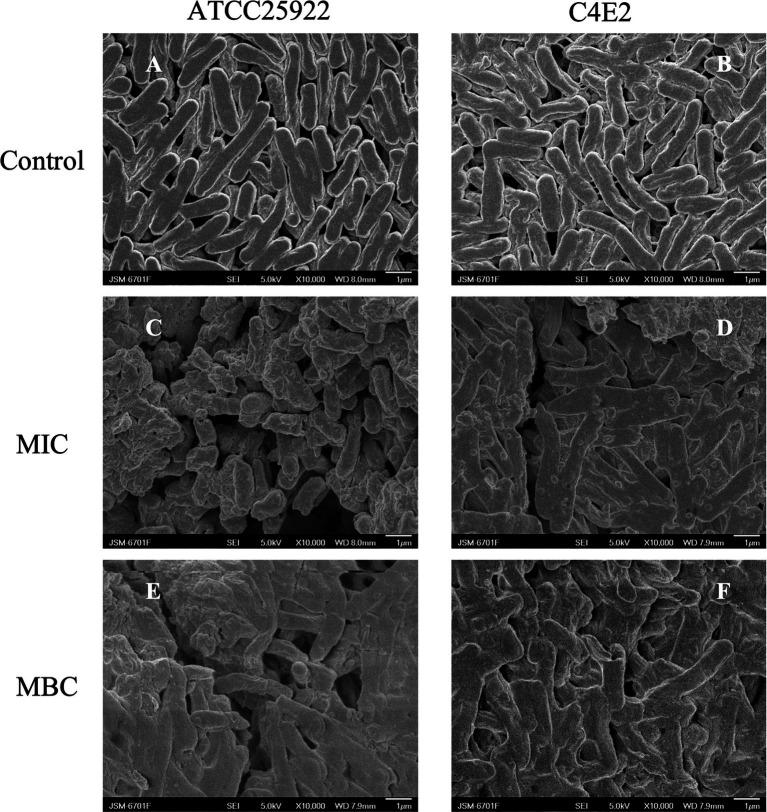
Disruption of cell envelope integrity by ICAC. Scanning electron microscopy (SEM) micrographs of the *E. coli* strains ATCC25922 and C4E2 after treatment with media alone **(A,B)**, at the MIC **(C,D)**, or at the MBC **(E,F)**. Magnification = 10,000×.

The ultrastructural changes in *E. coli* were examined via SEM. The cell membrane of *E. coli* ATCC25922 and C4E2 was an integrated and plump cell structure, and a typical rod-shaped morphology with a smooth surface was observed in the control group (Figures 3A,B). However, after 1 × MIC ICAC treatment, the membrane surfaces became irregular, folded and rough, and the cells were stacked and adhered (Figures 3C,D). Moreover, with the addition of MBC to the ICAC, severe damage and rupture of bacteria were observed. Moreover, the integrity of the bacteria was disrupted, causing the appearance of floccus, which led to more severe cell aggregation and overlap (Figures 3E,F).

SEM confirmed that ICAC induced severe physical damage and considerable morphological alterations in *E. coli*. These alterations may be due to ICAC enhancing cell permeability and destabilizing membrane integrity at the intracellular and outer membrane levels. Therefore, leakage of different cytoplasmic substances from the bacterial cells was further detected.

### 3.5 Cell wall permeability analysis

**Figure 4:**
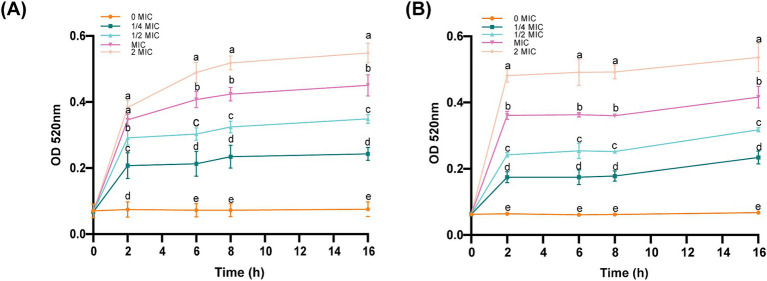
Effect of ICAC on the activity of *E. coli*. **(A)** ATCC25922; **(B)** C4E2. Different lowercase letters represent significant differences in ICAC treatment at different concentrations (*p* < 0.05). The results from all the experiments are presented as the means ± SDs of three replicates.

AKP is located between the cell wall and the cell membrane and could serve as an indicator of cell wall permeability. As shown in Figure 4, all the concentrations of the bacterial suspensions caused different degrees of increase in AKP compared to those in the control group. After the same incubation time, the concentration of ICAC was positively correlated with AKP leakage, suggesting that a high concentration of ICAC could significantly change the permeability of the cell wall and thus cause leakage of intracellular AKP and increase extracellular AKP activity.

### 3.6 OM permeability analysis

**Figure 5:**
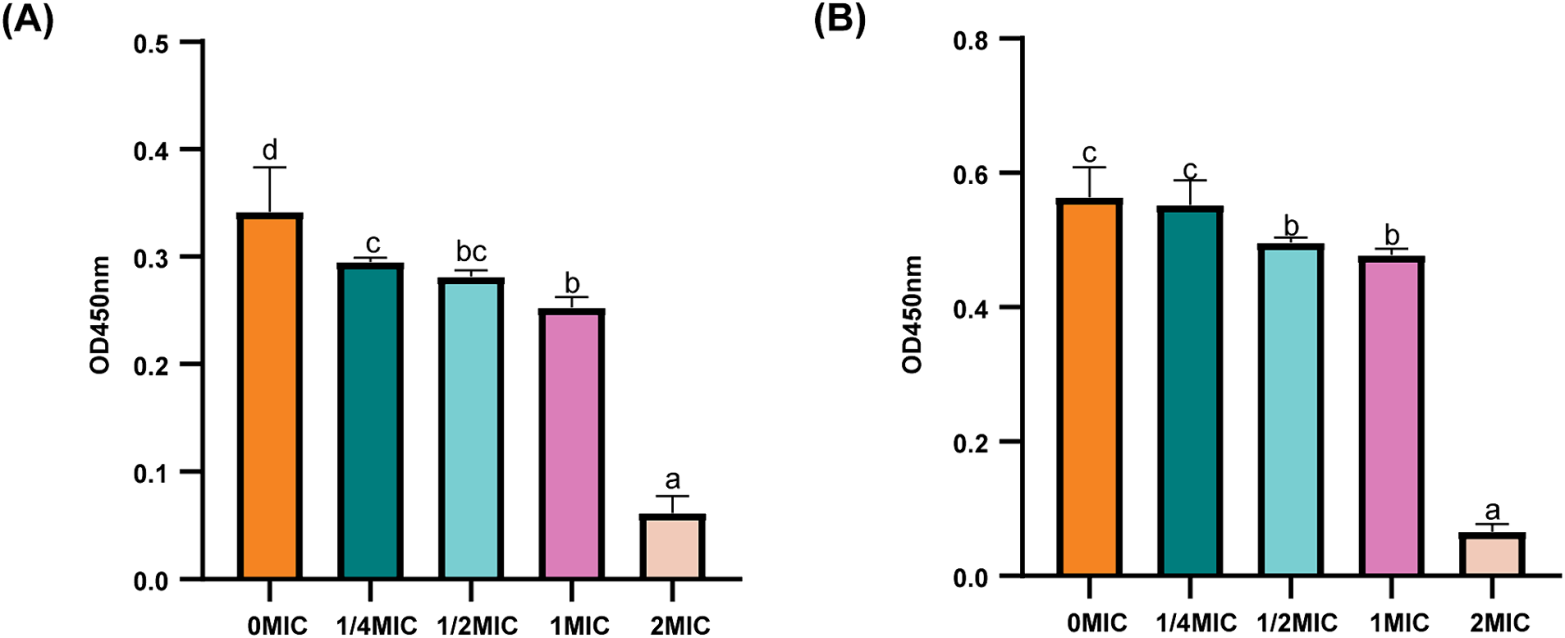
LPS contents of *E. coli* ATCC25922 **(A)** and C4E2 **(B)** treated with ICAC. Different lowercase letters represent significant differences in ICAC treatment at different concentrations (*p* < 0.05). The results from all the experiments are presented as the means ± SDs of three replicates.

LPS is the main component of OM in gram-negative bacteria. Therefore, the potential effects of ICAC on LPS-induced permeability were examined to investigate OM permeability. The results showed that the LPS content of *E. coli* significantly decreased (*p* < 0.05) after ICAC treatment in a dose-dependent manner (Figure 5). Therefore, ICAC could change the LPS concentration to increase the permeability of OM.

### 3.7 IM permeability analysis

**Figure 6:**
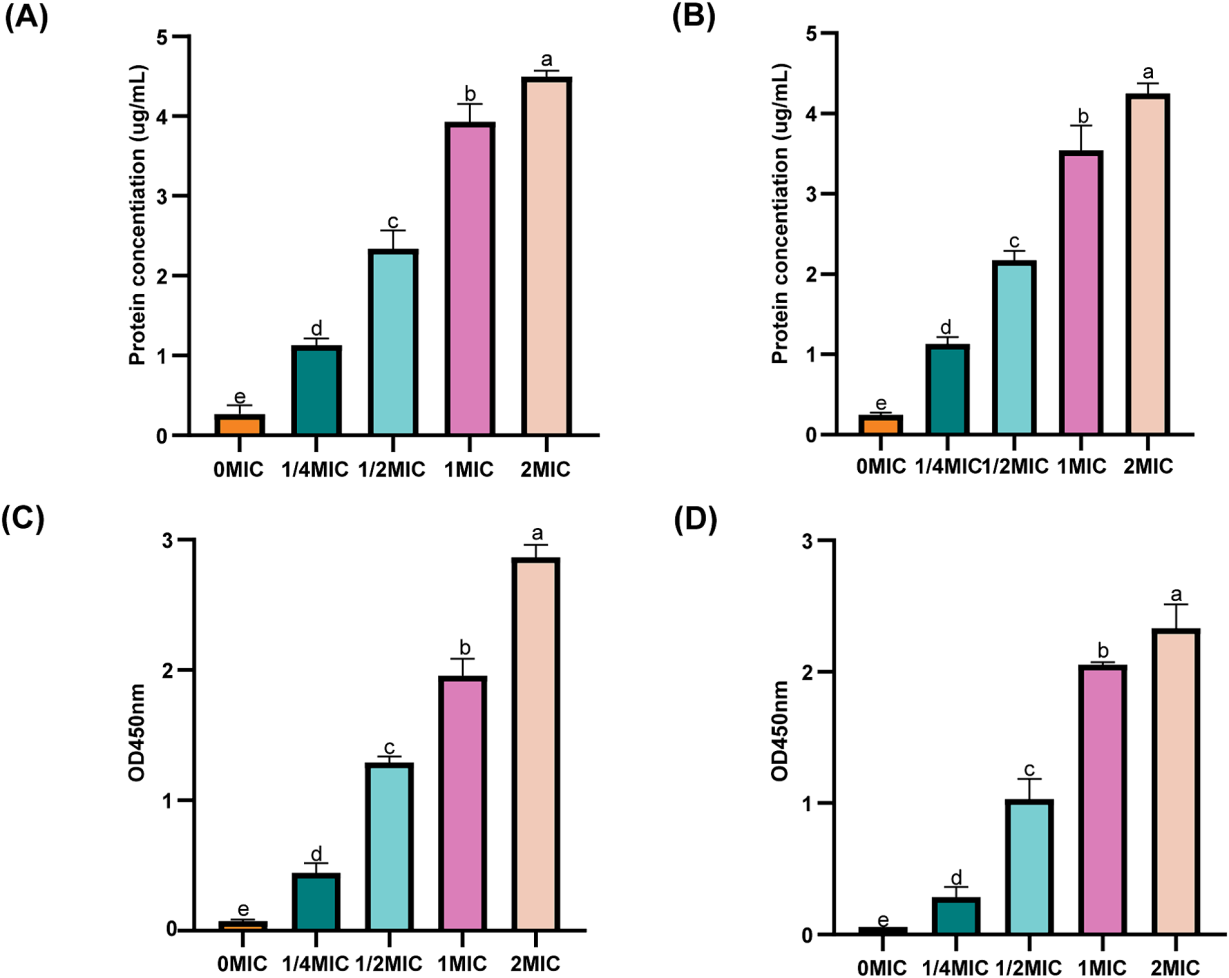
Effect of ICAC on the concentration of extracellular proteins and K^+^ concentration. **(A,B)** represent intracellular proteins released from ATCC25922 and C4E2, respectively. **(C,D)** represent K^+^ released from ATCC25922 and C4E2, respectively. Different lowercase letters represent significant differences in ICAC treatments at different concentrations (*p* < 0.05). Results from all experiments are presented as the means ± SDs of three replicates.

To investigate the effect of ICAC on the IM, the permeability of the membranes to intracellular proteins and K^+^ was monitored. As shown in Figure 6, compared to those in the control group (0 MIC), after incubation in 1/4 MIC, 1/2 MIC, 1 MIC and 2 MIC ICAC for 6 h, the extracellular protein and K^+^ release significantly increased with increasing ICAC concentration. The leakage of extracellular proteins and K^+^ from *E. coli* ATCC25922 and C4E2 sharply increased after treatment, suggesting that ICAC could act on the IM by increasing the permeability and release of intracellular contents.

### 3.8 IM integrity analysis

**Figure 7:**
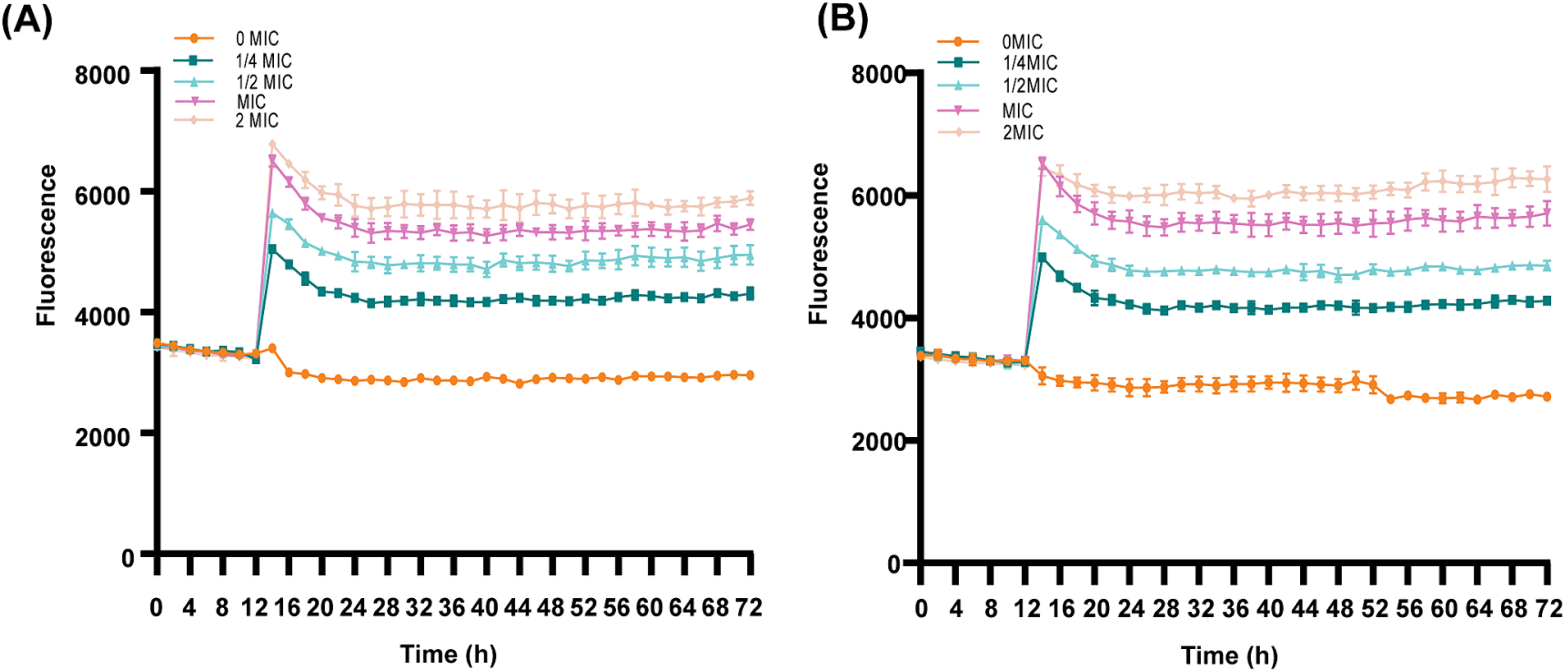
Dynamic fluorescence of PI-treated *E. coli* following the addition of ICAC at various concentrations. **(A,B)** ATCC25922 and C4E2, respectively. The results from all the experiments are presented as the means ± SDs of three replicates.

The cell membrane integrity of ICAC-treated bacteria was evaluated by the fluorescent probe cFDA and PI analysis. cFDA can penetrate into the intact cell membrane and be converted into carboxyfluorescein (cF), which emits green fluorescence. PI can only penetrate into injured cell membranes and emits red fluorescence upon staining DNA and RNA. Additionally, cells can emit yellow fluorescence when the membrane is damaged, but esterase activity also occurs. As shown in Figure 7 and Supplementary Table S1, after treatment with ICAC, the fluorescence intensity of PI significantly increased, and there was a positive correlation between the change in fluorescence and the ICAC concentration.

**Figure 8:**
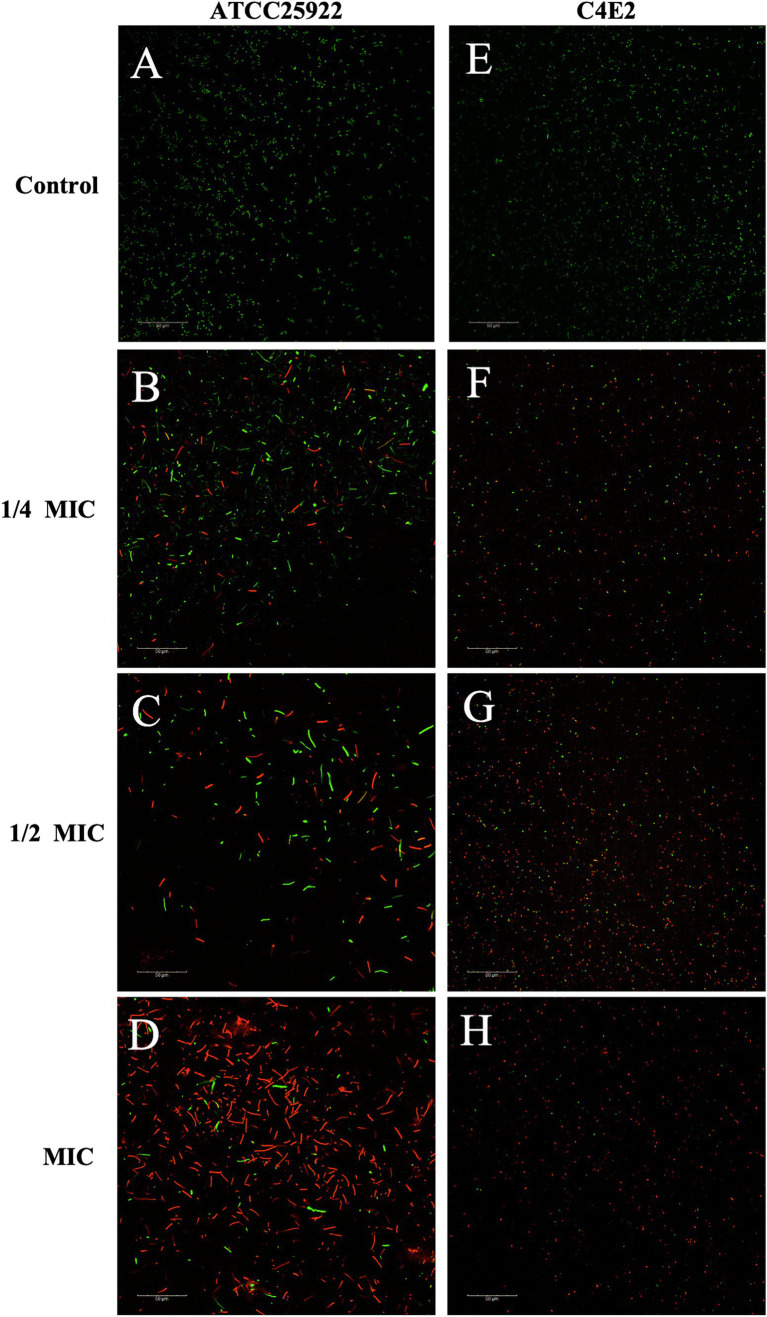
*E. coli* ATCC25922 and C4E2 treated with different concentrations 0**(A,E)**, 1/4**(B,F)**, 1/2**(C,G)**, 1**(D,H)** MIC of ICAC.

The results of the automatic microplate reader were verified by CLSM. The inner membrane of the untreated cells was not damaged, and the cells emitted green fluorescence (Figures 8A,E). However, the red fluorescence intensity of the *E. coli* cells increased with increasing concentrations of ICAC, suggesting that cell membrane integrity was disrupted (Figure 8). According to our results, green cells were still dominant after 1/4 MIC ICAC treatment, but the red fluorescent cells were more abundant as the ICAC concentration increased to 1 MIC. In addition, the ICAC-treated ATCC25922 cells no longer maintained their original shape and became longer. This may be because a compromised cell wall leads to bacterial expansion and deformation. The results of automatic microplate reader and CLSM analyses verified that the addition of ICAC further damaged the *E. coli* inner membrane in a concentration-dependent manner.

## 4 Discussion

The widespread emergence of multidrug-resistant bacteria and superbugs is still an enormous challenge for existing antimicrobial agents (Nang et al., 2021). The abuse and overuse of antimicrobial agents for the treatment of infectious diseases have significantly reduced the clinical efficacy of previously effective antimicrobial agents (Xu et al., 2022). Antimicrobial resistance and a lack of novel antimicrobial agents have made the treatment of bacterial infections increasingly difficult, and there is dire demand for finding new solutions to counter antibiotic-resistant organisms.

Natural plant compounds are potent antimicrobial agents with broad antibacterial spectra (Chiorcea-Paquim, 2023). The prevalence of new natural plant-derived compounds with outstanding antibacterial activity as alternatives to synthetic antimicrobial agents has increased. CAs are the most common hydroxycinnamate derivatives observed in the plant kingdom (Karaköse et al., 2011). Previous studies have reported that CA exhibits antibacterial effects against both gram-negative and gram-positive bacteria (Matias et al., 2019; Boukhibar et al., 2023).

In this study, six CA isomers were isolated from Stevia. Compared to the other isomers, ICAC exhibited the strongest antibacterial activity and widest range of antibacterial activity. *E. coli* is one of the major opportunistic pathogens causing bacterial infectious diseases and spreads resistance genes at full pace in people and animals (Henriot et al., 2022). The *in vitro* antibacterial activity results showed that ICAC had a good inhibitory effect on *E. coli* and that the antimicrobial activity was dose dependent.

Previous studies have shown that CA can play an antibacterial role by destroying the cell microstructure (Su et al., 2019). Our SEM images showed that ICAC could obviously destroy the micromorphology and microstructure of *E. coli* ATCC25922 and C4E2 cells. The gram-negative bacterial envelope is composed of a thin, rigid cell wall, a thick outer membrane and an inner membrane (Silhavy, 2015). The antimicrobial effects of ICAC were confirmed through cell structure, permeability and integrity analyses.

Based on the SEM results, we speculated that the change in micromorphology induced by ICAC was achieved through damage to the bacterial cell wall. The cell wall of *E. coli* is an elastic structure that provides permeability and physical protection and determines the shape of the cell (Gu et al., 2023). Natural compounds from traditional Chinese medicine containing CA possess antibacterial activity through destruction of the bacterial cell wall (He et al., 2018; Sun et al., 2020). In this study, we evaluated the permeability of the bacterial cell wall by measuring the AKP concentration. AKP leaks into the extracellular space by increasing the permeability of the bacterial cell wall (Wang et al., 2021). ICAC could induce the leakage of intracellular AKP and increase the activity of extracellular AKP, which indicated that the permeability of the cell wall of *E. coli* could be effectively disrupted by ICAC.

The OM of gram-negative bacteria mainly consists of LPS and lipoproteins, which are important for bacterial viability (Konovalova et al., 2016). LPS and lipoproteins in the OM are maintained together by electrostatic interactions with divalent cations required to stabilize the OM (Lou et al., 2011). CA, which is negatively charged, might bind to the OM by electrostatic interactions, disrupting the OM and leading to the loss of barrier function (Su et al., 2019; Sun et al., 2020). In the present study, the decrease in the LPS concentration indicated that ICAC could induce the exfoliation of LPS and disrupt the integrity of the OM.

Many antimicrobial agents containing CA can target the IM to increase its permeability and release small molecules, which seriously affects the metabolism of the bacterium (Yi et al., 2010; Zheng et al., 2016; Gu et al., 2023). Our results suggested that significant leakage of intracellular protein and K^+^ from *E. coli* ATCC25922 and C4E2 occurred after ICAC treatment. The bacterial IM provides a permeability barrier for the passage of small ions, and the increased release of K^+^ indicates that this permeability barrier is disrupted. Leakage of bacterial intracellular macromolecules also suggested that ICAC increases IM permeability.

Living cells with esterase activity emit green when cFDA permeates into the cells (Sun et al., 2020). PI is a fluorescent dye that has a high affinity for DNA but is impermeable to the integrity of the cell membrane. However, PI is commonly used as a viability probe because it can enter injured membranes or dead cells and react with DNA to emit red fluorescent signals (Müller et al., 2016; Kannan et al., 2019). Therefore, the fluorescence conferred by a probe is generally associated with damage to membrane integrity. Our study provided evidence that ICAC disrupted IM integrity, resulting in an increase in the fluorescence intensity of ICAC-treated cells, and that the damage to the IM was significantly enhanced (*p* < 0.05) with increasing ICAC concentration. Additionally, the CLSM images indicated that the number of cells with red fluorescence increased and the amount of green fluorescence decreased as the concentration of ICAC increased, which was also consistent with the automatic microplate results.

In conclusion, ICAC potently inhibited *E. coli* microstructure permeability and integrity, ultimately resulting in cell death. Based on the experimental data, ICAC killed bacteria by provoking irreversible permeability changes in the IM and inducing the exfoliation of the OM. Moreover, ICAC could target bacterial cell walls and disrupt the ability to maintain cell wall permeability and morphology. Our findings in this study successfully suggest the possibility that ICAC may be used to treat human infections caused by antibiotic-resistant pathogenic *E. coli*.

## Data Availability

The original contributions presented in the study are included in the article/Supplementary material, further inquiries can be directed to the corresponding authors.
