# Supplementary material for: Antibacterial activity and mechanism of Stevia extract against antibiotic-resistant Escherichia coli by interfering with the permeability of the cell wall and the membrane
Source: Front Microbiol. 2024 Sep 18;15:1397906. doi: 10.3389/fmicb.2024.1397906 (PMC11445074; doi:10.3389/fmicb.2024.1397906)
Supplement: Supplementary file 1 [file Data_Sheet_1.docx]

**Supplementary Material**

**Table. S1 The fluorescence values of PI under different ICAC treatment times.**

| Concentration | ATCC25922 | | | C4E2 | | |
| --- | --- | --- | --- | --- | --- | --- |
|  | 0min | 36min | 72min | 0min | 36min | 72min |
| 0 MIC | 3490.33±33.25^a^ | 2869.67±12.90^a^ | 2957.33±39.55^a^ | 3381.33±42.03^ab^ | 2926.33±124.44^a^ | 2714.67±48.40^a^ |
| 1/4 MIC | 3477.00±42.04^a^ | 4187.33±87.89^b^ | 4309.33±103.76^b^ | 3449.67±48.21^a^ | 4165.00±68.20^b^ | 4280.67±27.06^b^ |
| 1/2 MIC | 3451.33±21.55^ab^ | 4792.33±121.26^c^ | 4947.00±163.68^c^ | 3400.33±37.86^ab^ | 4771.00±59.63^c^ | 4856.67±77.18^c^ |
| 1 MIC | 3424．33±10.51^b^ | 5309.67±127.30^d^ | 5450.00±85.85^d^ | 3436.33±30.50^ab^ | 5546.33±146.12^d^ | 5710.00±198.73^d^ |
| 2 MIC | 3424.67±10.26^b^ | 5779.00±219.58^e^ | 5887.33±115.63^e^ | 3407.93±45.45^b^ | 5954.33±59.01^e^ | 6269.67±207.28^e^ |


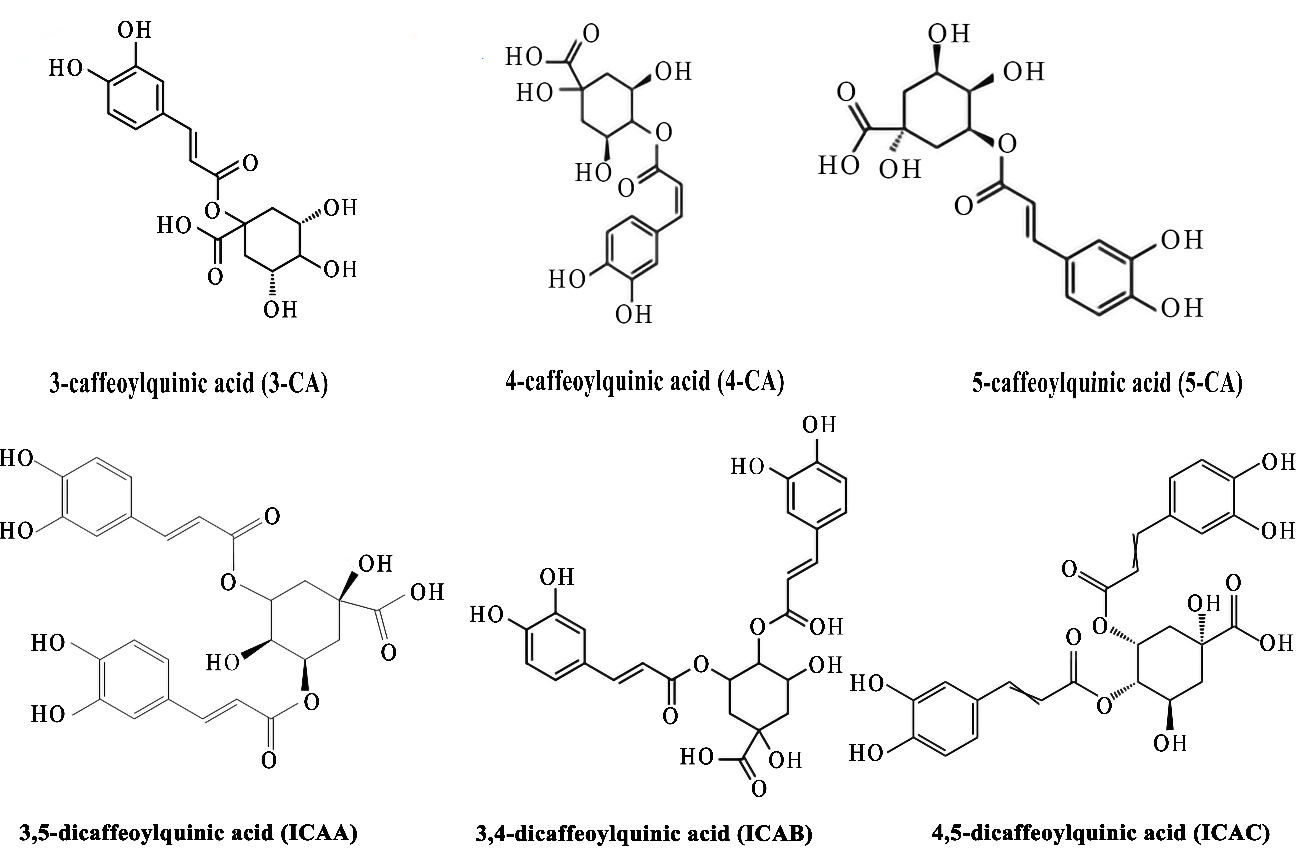


**Figure. S1 Structures and numberings of the chlorogenic acid isomers found in stevia.**
